# The research trends of macrophages in acute kidney injury: A bibliometric analysis

**DOI:** 10.1097/MD.0000000000046735

**Published:** 2025-12-26

**Authors:** Xiangyu Zhang, Qiao Zhou, Sizeng Bao, Chengmin Ma, Hanlu Wang, Lingling Bao, Jianwei Ma, Yanping Wang, Jiancheng Huang, Xueyan Bian

**Affiliations:** a Department of Nephrology, The First Affiliated Hospital of Ningbo University, Ningbo, China; b Department of Nephrology, Huaian Hospital Affiliated to Yangzhou University (The Fifth People’s Hospital of Huaian), Huaian, China; c Department of Emergency, The First Affiliated Hospital of Ningbo University, Ningbo, China; d Harvard Center for Polycystic Kidney Disease Research and Renal Division, Brigham and Women’s Hospital, Harvard Medical School Boston, Boston, MA.

**Keywords:** acute kidney injury, bibliometric analysis, CiteSpace, ferroptosis, macrophages, systemic inflammation

## Abstract

**Objective:**

In recent years, the study of macrophages has gained prominence as a promising avenue for understanding the pathogenesis and therapeutic prospects of acute kidney injury (AKI). In this work, we utilize a bibliometric analysis to map the existing research landscape concerning macrophages in the context of AKI. We aim to provide valuable insights and scholarly references that can facilitate the advancement of comprehensive research and innovation in this field. Our goal is to provide valuable knowledge and academic sources that support the progress of thorough research and breakthroughs in connected fields.

**Methods:**

We incorporated studies focused on macrophages in AKI that were retrieved from the Web of Science Core Collection. All publications were exported in plaintext full-record format and subsequently analyzed using CiteSpace 6.4.R1 for bibliometric evaluation.

**Results:**

A total of 1483 records meeting the inclusion criteria were analyzed. The number of publications in the 1st 12 years was relatively low, but there was a notable increase in publications in 2019 and 2021. The research, encompassing contributions from 56 countries and 437 institutions, is led by China and US Department of Veterans Affairs. The authors with the highest number of publications are Hans-Joachim Anders and Mark D. Okusa, and Bonventre JV was the most frequently cited author. The journal with the highest number of co-citations is Kidney International. Prominent keywords in the literature include macrophages, inflammation, and extracellular vesicles. Keywords extracted from the analysis mainly focus on AKI, expression, acute renal failure, activation, inflammation, and macrophages.

**Conclusion:**

This research provides a comprehensive bibliometric evaluation, thereby enhancing our understanding of the current state of macrophage studies in AKI. As a result, it assists both experienced researchers and newcomers by facilitating quick access to crucial information and promoting the extraction of innovative concepts within this specialized field.

## 1. Introduction

Acute kidney injury (AKI) is a condition characterized by fast deterioration in renal function, and its high morbidity and mortality have attracted the attention of an increasing number of individuals.^[1]^ Various forms of unknown etiology acute renal damage, with varying prognoses, have a complicated pathophysiological process.^[2,3]^ Currently, renal tubular epithelial cell (TEC) damage or inability to adjust good repair, immune-mediated inflammation, microcirculation dysfunction, and other pathological physiological or metabolic processes have been proven to be closely involved in the occurrence of AKI in the process of development.^[4,5]^ It is widely accepted that injury to renal TECs is the primary factor in the development of AKI. Furthermore, macrophages (M), derived from common myeloid progenitor cells in the bone marrow, play pivotal roles in the pathogenesis and progression of AKI. Their involvement is particularly critical during the transition from AKI to chronic kidney disease (CKD), where they contribute to both tissue injury and repair processes.^[6]^ Based on their functions and phenotypes, macrophages can be classified into 2 main types: M1 type and M2 type. M1-type macrophages are usually considered pro-inflammatory, while M2-type macrophages have anti-inflammatory and tissue repair functions.^[7]^ Importantly, macrophage phenotypes exhibit dynamic shifts throughout the stages of renal injury. These phenotypic changes modulate macrophage interactions with other renal cell types via multiple mechanisms, including the release of soluble factors and extracellular vesicles (EVs), as well as direct cell contact.^[8,9]^ These interactions are crucial for the transition from AKI to CKD, as they may either promote healing or exacerbate injury, depending on the phenotype of macrophages and the signals they receive from the environment.^[10]^

Prior research has shown that the primary function of macrophages is to protect the host and remove dying cells.^[11]^ However, an increasing number of studies have demonstrated that macrophages have diverse functions in tissue growth, maintenance of stability, and healing of wounds.^[12]^ There is a growing recognition among people that macrophages play a crucial role in tissue repair, regeneration, and the formation of fibrosis. Macrophages are capable of creating several growth factors that stimulate cell proliferation and facilitate healing.^[13]^ Recent research has discovered that immune cells, particularly macrophages, have a crucial and intricate function in AKI.^[14,15]^ Researchers are currently achieving many important results in this field. Macrophage polarization into pro-inflammatory (M1) and anti-inflammatory (M2) phenotypes is a crucial determinant of outcomes in AKI. Notably, activation of the retinoic acid signaling pathway balances these macrophage phenotypes, mitigates macrophage-mediated damage, and promotes tissue repair. Although suppressed under normal conditions in adults, this pathway reactivates following AKI, playing a key role in injury mitigation and repair by modulating macrophage activity.^[16]^ Evidence indicates that macrophage polarization dynamically shifts during the progression of AKI. Initially, M1 macrophages predominate in the early injury phase; subsequently, M2 macrophages increase with disease progression, contributing to inflammation resolution and tissue repair.^[17]^ Consequently, modulating macrophage polarization represents a potential therapeutic strategy for AKI. Furthermore, specific signaling pathways, such as Ccl5/NF-κB, are key mediators of macrophage-driven inflammation in AKI. Notably, tubule-derived long non-coding RNAs, including lncRNA-gm33782, can exacerbate renal inflammation and injury during AKI by activating M1 macrophage polarization via the Ccl5/NF-κB pathway. These findings highlight the therapeutic potential of targeting such pathways to modulate the immune response and improve AKI outcomes.^[18]^

Bibliometric analysis is an interdisciplinary science that offers insights into scientific development trends and informs the direction of future research through the quantitative analysis of bibliometric data using mathematical and statistical methods.^[19]^ Traditional literature reviews often encounter significant challenges in objectively synthesizing the extensive body of research across various fields and sub-fields. Bibliometric analysis presents a robust alternative by leveraging its capacity to process large-scale datasets, thereby enabling a systematic and comprehensive evaluation of tens of thousands or even millions of academic publications. Moreover, it facilitates the visualization of collaborative networks, which aids in identifying regional and institutional research disparities. Furthermore, bibliometrics enables the timely identification of emerging research frontiers and hotspots through temporal analysis (such as detecting burst terms, tracking surges in newly published literature, and analyzing highly cited recent works) thus offering valuable insights into evolving research trends and dynamic scholarly domains.^[20,21]^ Currently, bibliometric analysis has been extensively applied in various domains, including medicine, biology, physics, and other natural sciences, as well as social science disciplines such as sociology and economics.^[22–24]^ Increasing empirical evidence indicates that bibliometric analysis plays a crucial role in helping future researchers rapidly and comprehensively understand a given research field, thereby laying a solid foundation for their scholarly endeavors.

In this study, we aim to conduct a comprehensive bibliometric analysis to evaluate the published literature and contributing entities in the field of macrophages and AKI research. Our objectives include providing a visual representation of the research landscape, highlighting key terms, authors, countries, and institutions, and identifying research hotspots and emerging frontiers in this field.

## 2. Materials and methods

### 2.1. Data source

**Figure 1.**
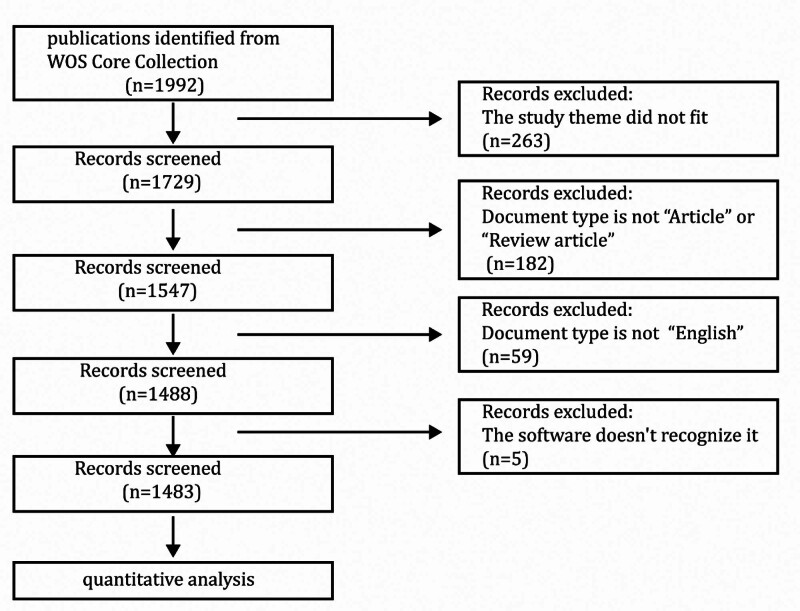
Flow diagram of literature selection.

The literature on macrophages in AKI was screened using the Web of Science (WoS) Core Collection database. The search strategy employed the keywords TS = (“macrophage” OR “macrophages”) AND TS= (“acute kidney injury” OR “acute kidney failure” OR “acute renal injury” OR “acute renal failure” OR “AKI”). The timeframe for the search was set from January 1, 2004, to December 31, 2024, yielding a total of 1992 articles. Only research articles and review papers published primarily in English were retained, resulting in 1483 articles included in the study. The process of screening articles is depicted in Figure 1. The documents were then selected as full records and their references were cited before being exported in.txt format for further analysis.

### 2.2. Data extraction and analysis

Using CiteSpace, we conducted a visualization analysis of collaboration networks among countries, institutions, journals, and authors, as well as co-citation clustering of keywords. By examining keyword frequency, centrality, and prominence, we can comprehensively understand the current research status, hotspots, and development trends in this field.^[25–27]^ This study focuses on literature related to macrophages in AKI. First, a preliminary statistical analysis of the retrieved literature was conducted to obtain basic data on macrophages in AKI research. Subsequently, using CiteSpace (version 6.4.R1; Chaomei Chen College of Computing and Informatics, Drexel University, Philadelphia), we visually presented information on core authors, document co-citation, disciplinary distribution, and geographical distribution in this field. Finally, we performed an in-depth study of the visualization results in conjunction with the content of the literature, analyzing sample data to calculate information on citation counts, authors, and geographical aspects. This analysis helps to elucidate the research hotspots and significant research findings in this field, further uncovering the knowledge base and research trends in the domain of macrophages in AKI.

## 3. Results

### 3.1. Posting trends

**Figure 2.**
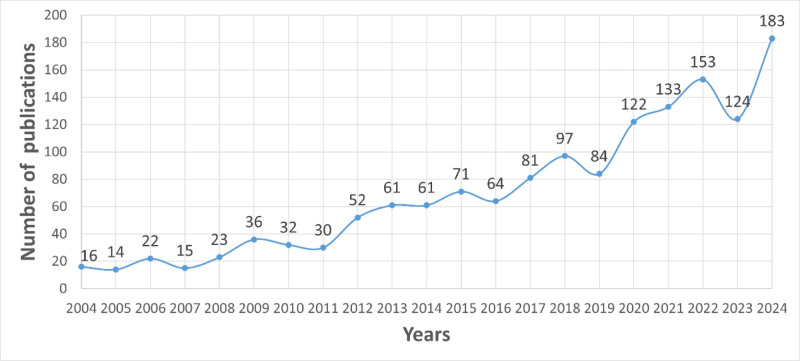
The number of publications by year over the past 20 years.

From 2004 to 2024, 1483 records were identified from the database, resulting in an average annual publication count of 70.6, as illustrated in Figure 2. During the period from 2004 to 2018, the annual output remained below 100, indicating a slow developmental phase. Beginning in 2012, the annual number of relevant scholarly publications exceeded 50, peaking at 183 in 2024. Interestingly, there has been a decline in the number of published works since 2022, suggesting a potential waning interest in the study of macrophages in AKI. However, the number of published works experienced a rapid increase once again in 2023. Whether this trend will continue to grow in the future requires further observation.

### 3.2. Distribution of countries

**Figure 3.**
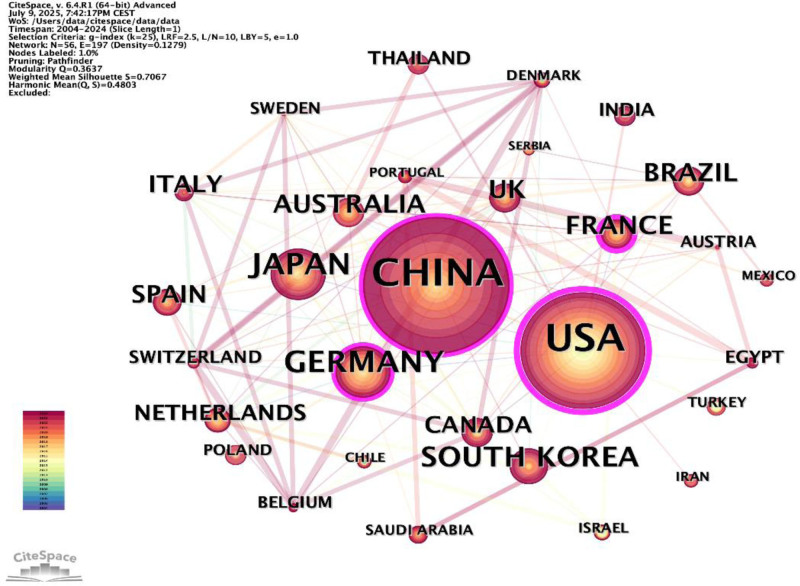
Country/region network visualization.

Our data analysis reveals that China is the foremost country in terms of the number of published studies in this field, with 533 publications. This is followed by the United States, which has 457 publications, and Japan, with 129 publications (Fig. 3). Table 1 lists the top 10 countries or regions with the highest number of publications. China, the United States, Germany, and Japan play crucial roles in this field and maintain relatively close collaborative relationships with other countries. Additionally, it is observed that research activities in China have significantly increased in recent years. However, there remains room for improvement in the quality of Chinese research.

**Table 1:** Publications in the 10 most productive countries/regions.

Rank	Country	Count	Centrality	Year
1	China	533	0.15	2004
2	USA	457	0.49	2004
3	Japan	129	0.04	2004
4	Germany	124	0.2	2005
5	South Korea	86	0.02	2006
6	UK	57	0.06	2004
7	France	53	0.23	2006
8	Australia	53	0.07	2004
9	Brazil	51	0.02	2005
10	Spain	47	0	2006

### 3.3. Institutional cooperation network

**Figure 4.**
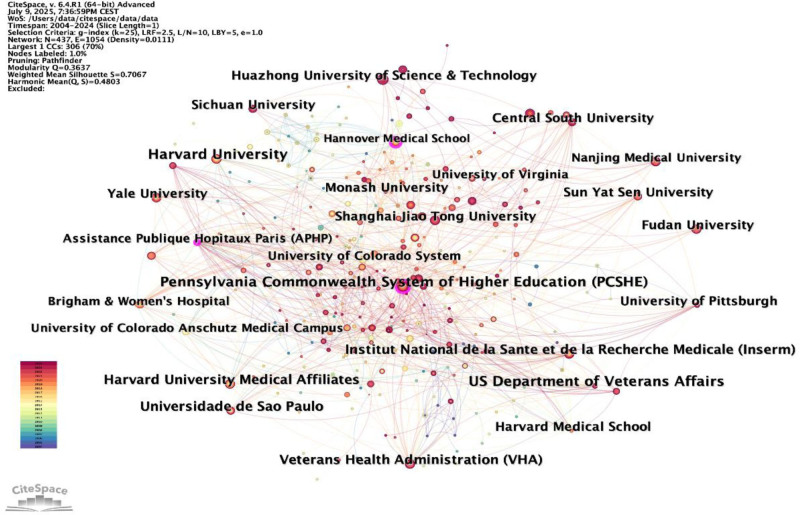
CiteSpace-generated institutional collaboration visualization mapping.

In Figure 4, we utilized CiteSpace software to analyze the 437 institutions that contributed to the literature included in this study. The results indicate that US Department of Veterans Affairs is the leading research institution in terms of the number of publications (37 papers), followed by the Pennsylvania Commonwealth System of Higher Education (PCSHE) (35 papers) and Harvard University (34 papers). Additionally, Table 2 lists the top 10 institutions by publication volume. We found that the publishing institutions are primarily comprehensive universities and medical schools. Notably, the US Department of Veterans Affairs holds a significant position in this field.

**Table 2:** Publications in the 10 most productive institutions.

Rank	Country	Count	Centrality	Year
1	US Department of Veterans Affairs	37	0.05	2005
2	Pennsylvania Commonwealth System of Higher Education (PCSHE)	35	0.12	2005
3	Harvard University	34	0.04	2006
4	Veterans Health Administration (VHA)	33	0.08	2005
5	Harvard University Medical Affiliates	33	0.04	2006
6	Institut national de la santé et de la recherche médicale (Inserm)	31	0.05	2006
7	Universidade de São Paulo	30	0.03	2005
8	University of Pittsburgh	26	0.05	2005
9	Fudan University	25	0	2004
10	Sichuan University	24	0.02	2018

### 3.4. Keyword network analysis

#### 3.4.1. Co-occurrence network of keyword

**Figure 5.**
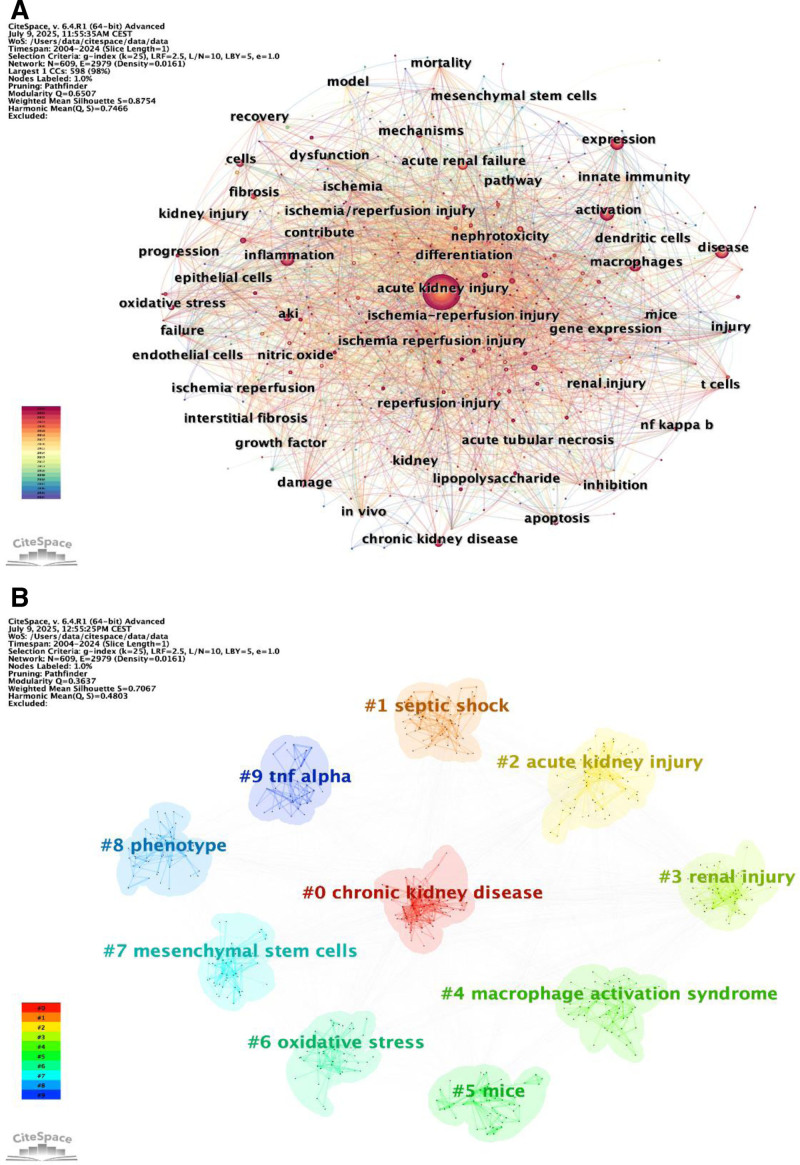
Keyword co-occurrence network graph generated by CiteSpace. (A) Keyword time overlay visualization. (B) Keyword co-occurrence map.

Keywords in a paper are the “eyes” of the document, providing a concise and insightful summary of its content. By statistically analyzing the frequency of keywords in a paper, high-frequency keywords can be identified, which to some extent represent the research hotspots in that particular field or topic. CiteSpace was utilized for visual analysis, generating keyword co-occurrence network diagrams and overlay visualization diagrams, which are shown as follows: AKI, expression, acute renal failure, activation, inflammation, and macrophages (Fig. 5A and B).

#### 3.4.2. Keyword emergence network

**Figure 6.**
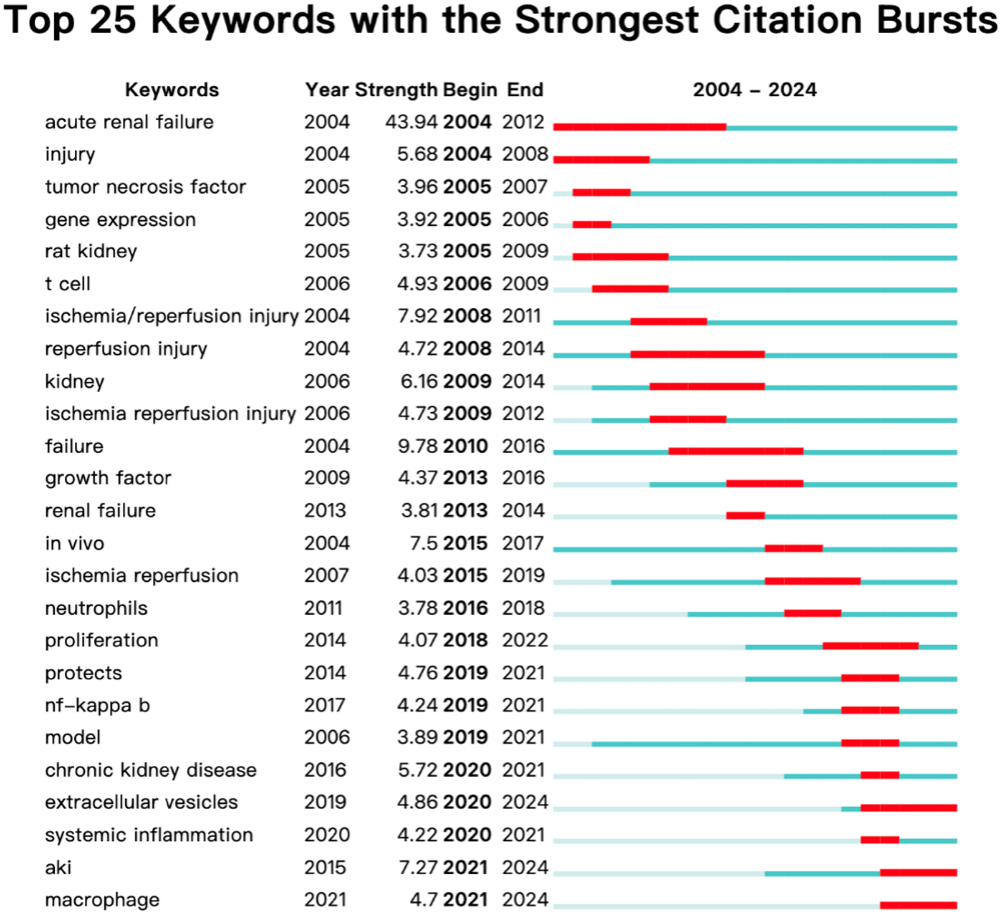
CiteSpace visualization map of top 25 keywords with the strongest citation bursts involved in macrophages in AKI. AKI = acute kidney injury.

Keyword emergence denotes a significant increase in the frequency of keywords over a short duration. Familiarity with current research trends can assist in identifying the most pertinent and innovative fields of study. Figure 6 displays the keyword emergence analysis of macrophages in AKI research literature. The evidence suggests that the research in this particular field began in 2004, and a growing number of related studies surfaced in 2012.

The primary focus of research centered on kidney injury and kidney failure. Moreover, from the beginning, the emphasis of the study was on in vivo and in vitro experiments investigating acute renal failure. The research hotspots in recent years were predominantly on inflammation, EVs, and macrophages. “Systemic inflammation,” “NF-κB,” “EVs,” and “chronic kidney disease” appeared later and were noticed later. The keywords with the strongest cited outbreaks were even acute renal failure (strength = 43.94), failure (strength = 9.78) and ischemia/reperfusion injury (strength = 7.92). Systemic inflammation is the keyword with stronger outbreaks that appeared in 2020, which could be a hotspot for research or a turning point with prospective research implication.

#### 3.4.3. Keyword time-zone view

**Figure 7.**
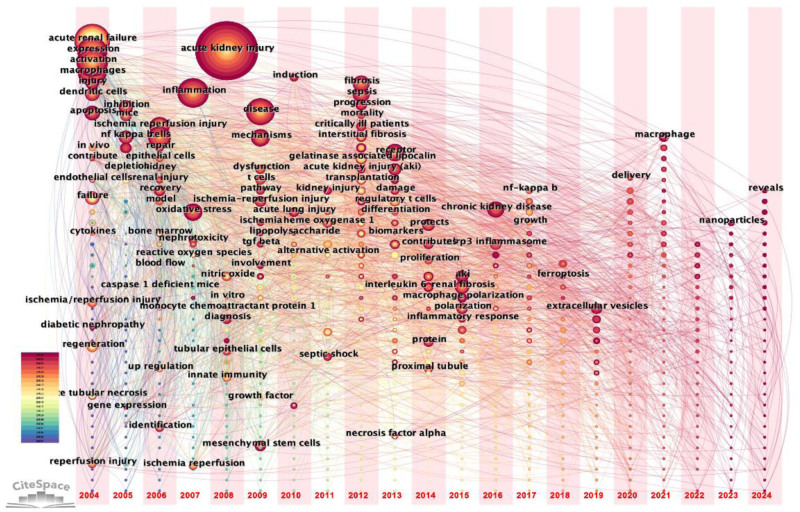
Keyword time-zone diagram.

The analysis of keyword fluctuations over time can yield valuable insights into the evolution of research topics and the emergence of new areas of focus. Figure 7 presents a chronological representation of terms that appear more than once, spanning the period from 2004 to 2024. This figure arranges each term within each time bar according to its initial appearance, thereby illustrating the progression of keywords and their interrelationships.

The keywords that appeared from 2004 to 2008 include: “expression,” “activation,” “acute kidney injury”; “acute renal failure”; “ischemia–reperfusion injury”; “inflammation”; “oxidative stress,” from 2009 to 2013: “dysfunction,” “mechanisms,” “t cells,” “interstitial fibrosis,” “transplantation,” “sepsis”; “differentiation”; “receptor”; from 2014 to 2018: “chronic kidney disease,” “proximal tubule,” “protein,” “macrophage polarization”; “ferroptosis,” “inflammatory response,” from 2019 to 2024: “extracellular vesicles,” “delivery”; “macrophage,” “nanoparticles,” “reveals.”

#### 3.4.4. Keyword timeline diagram and ridge plot

**Figure 8.**
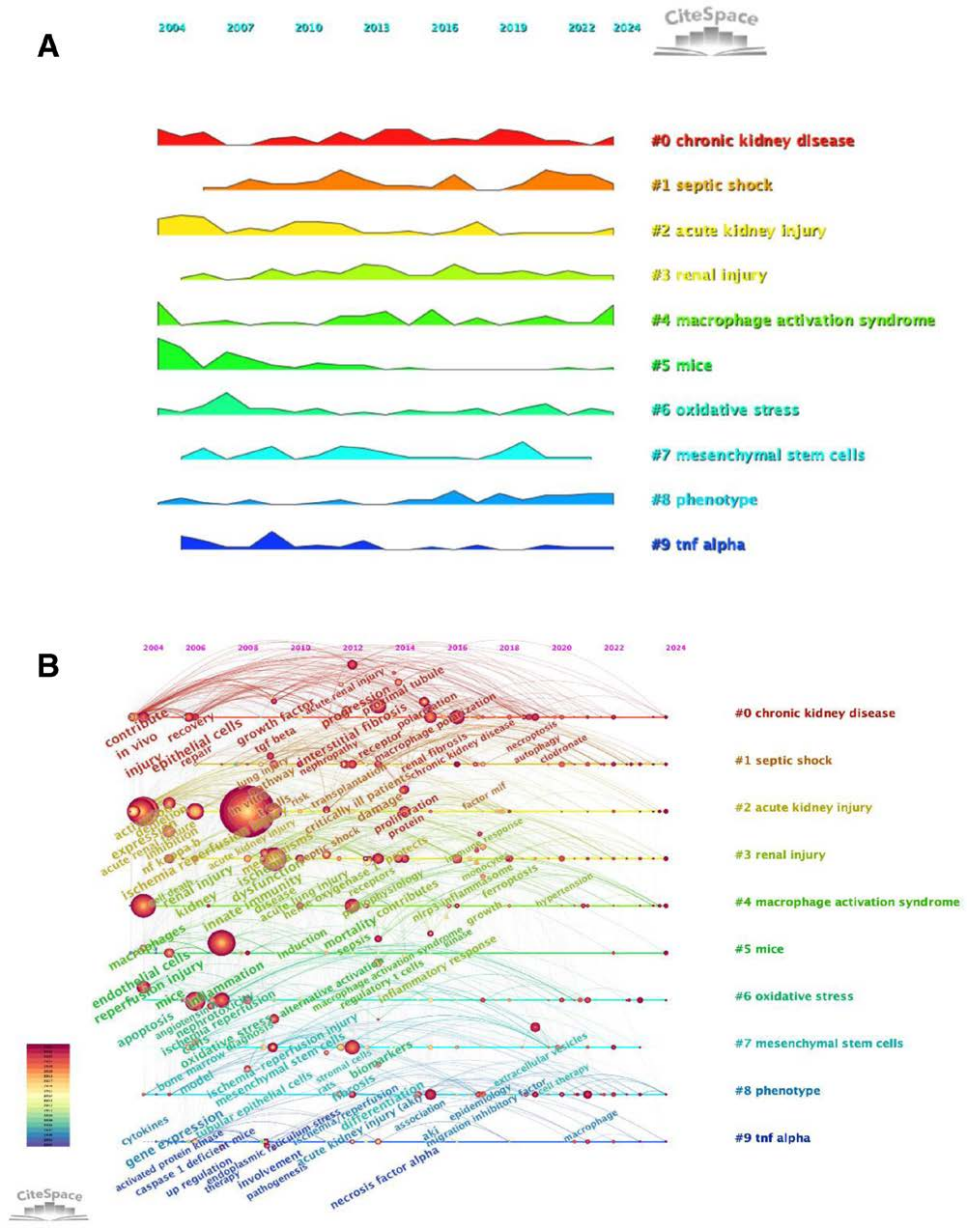
Keyword timeline diagram and ridge plot. (A) Ridge plot diagram. (B) Keyword timeline diagram.

We performed an analysis on the clustering of keywords depicted in Figure 5B, as well as the sequential pattern of their recurrence, to construct a ridgeline plot (Fig. 8A). From 2004 to 2012, there was significant interest in CKD, apoptosis, inflammation, AKI, and t cells. Following 2012, macrophage polarization, ferroptosis, EVs, and proliferation received more focus. Furthermore, as depicted in Figure 8B, we observed a gradual decline in research interest in clusters #7 (mesenchymal stem cells), while sustained research interest remains for clusters #0 (chronic kidney disease), #1 (septic shock), #2 (acute kidney injury), #3 (renal inhury), #4 (macrophage activation syndrome), #5 (mice), #6 (oxidative stress), #8 (phenotype), and #9 (tnf alpha). The current research hotspots in this field also corroborate this observation.

### 3.5. Distribution by journal

**Figure 9.**
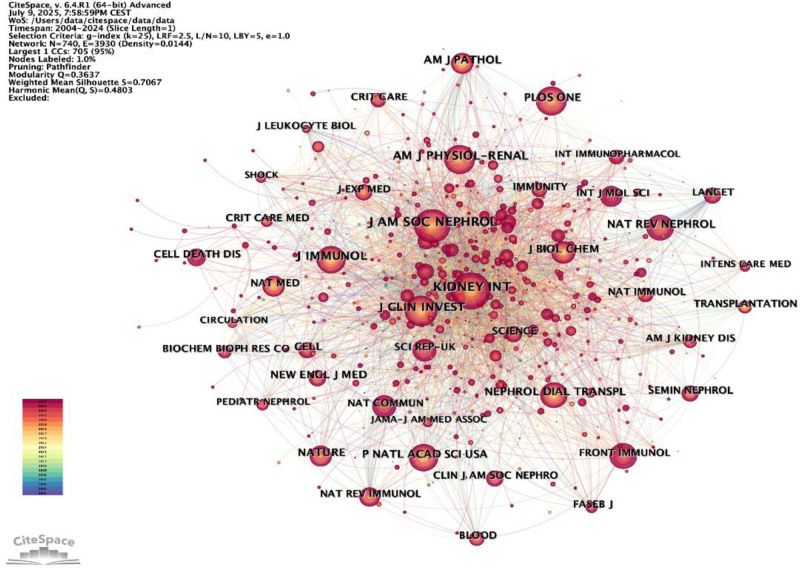
Density visualization map of journal citations.

The journals that have had the most significant impact on the development of this field were identified through co-cited journal analysis. The size of a node is determined by the number of citations it has received, while the width of a link reflects the strength of the connection between 2 nodes. In total, articles on macrophages and AKI have been published across 740 journals (Fig. 9). The journals with the highest number of citations are listed in Table 3, highlighting the top 10. Kidney International ranks 1st in terms of the number of publications (1207), followed by the Journal of the American Society of Nephrology (1139) and the Journal of Clinical Investigation (911). All of this can provide important submission information for researchers.

**Table 3:** The top 10 most citation journals in the field of macrophages in AKI.

Rank	Journal	Count	Centrality	Year	JCR
1	Kidney Int	1207	0.01	2004	Q1
2	J Am Soc Nephrol	1139	0.01	2004	Q1
3	J Clin Invest	911	0.01	2004	Q1
4	Am J Physiol Renal	868	0	2004	Q1
5	J Immunol	702	0	2004	Q2
6	P Natl Acad Sci USA	648	0	2004	Q1
7	Nephrol Dial Transpl	641	0	2004	Q1
8	Plos One	594	0	2010	Q1
9	J Biol Chem	528	0	2004	Q1
10	Nat Rev Nephrol	507	0	2010	Q1

AKI = acute kidney injury.

### 3.6. Document co-citation network

**Figure 10.**
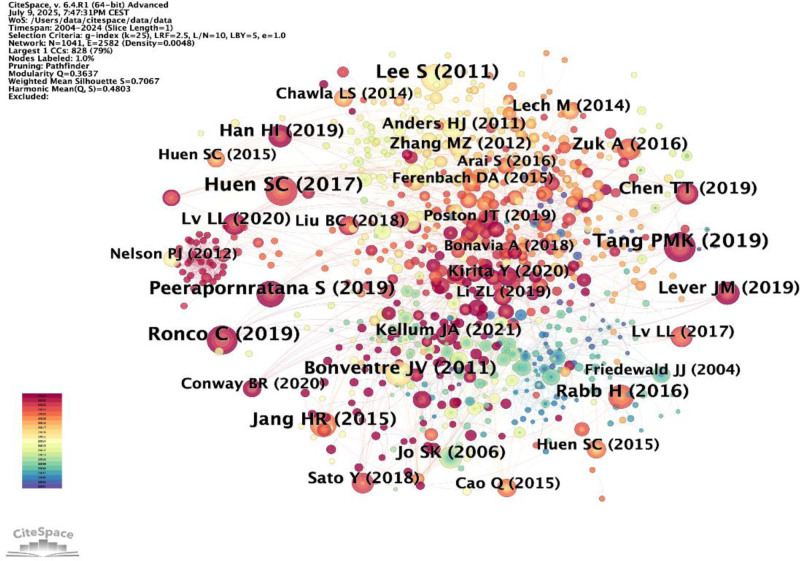
CiteSpace-generated co-cited references visualization mapping.

We utilized CiteSpace to analyze co-cited references, as illustrated in Figure 10. From the 1041cited pieces of literature, we meticulously examined the citations that ranked in the top 10 based on co-citation frequency, as detailed in Table 4. Notably, 3 of these articles were published before 2016, while the remaining 7 were published between 2016 and 2019. Remarkably, the study conducted by Tan PKM et al emerged as the most cited, with 60 citations, and an additional 6 papers received over 40 citations each.

**Table 4:** Top 10 articles with the most citations in the field of macrophages associated with AKI.

Rank	Author	Title	Citations	Year
1	Tan PMK	Macrophages: versatile players in renal inflammation and fibrosis	60	2019
2	Huen SC	Macrophages in renal injury and repair	55	2017
3	Ronco C	Acute kidney injury	53	2019
4	Lee S	Distinct macrophage phenotypes contribute to kidney injury and repair	52	2011
5	Peerapornaratana S	Acute kidney injury from sepsis: current concepts, epidemiology, pathophysiology, prevention and treatment	46	2019
6	Jang HR	Immune cells in experimental acute kidney injury	43	2015
7	Bonventre JV	Cellular pathophysiology of ischemic acute kidney injury	43	2011
8	Rabb H	Inflammation in AKI: current understanding, key questions, and knowledge gaps	38	2016
9	Han HI	The role of macrophages during acute kidney injury: destruction and repair	35	2019
10	Lever JM	Resident macrophages reprogram toward a developmental state after acute kidney injury	35	2019

AKI = acute kidney injury.

The top 10 most cited references include 8 reviews and 2 research articles. The majority of the top 10 co-cited research primarily delved into exploring the mechanisms underlying the initiation of AKI, with particular emphasis on the role of inflammation and macrophage activation in AKI. Among them, Tan PMK’s article is the most cited work. As demonstrated by Tan PMK et al, macrophages play a crucial role in immune surveillance and the maintenance of renal homeostasis, with their response to renal injury varying significantly according to the type and duration of the injury. Macrophages recruited from the bone marrow can directly transform into myofibroblasts in the injured kidney. This process, known as macrophage-to-myofibroblast transition (MMT), is mediated by TGF-β1-Smad3 signaling via a Src-centric regulatory network. MMT may represent a critical checkpoint whereby chronic inflammation progresses to pathological fibrosis. This reflects the growing body of recent research focusing on the role of macrophages in renal inflammation and fibrosis. Moreover, Huen SC’s research focuses on the latest developments in understanding the function of macrophages in kidney damage. Huen SC et al specifically highlight how monocyte recruitment and macrophage activation are influenced, as well as the crosstalk between macrophages and renal parenchymal cells.

### 3.7. Analysis of the most influential authors

**Figure 11.**
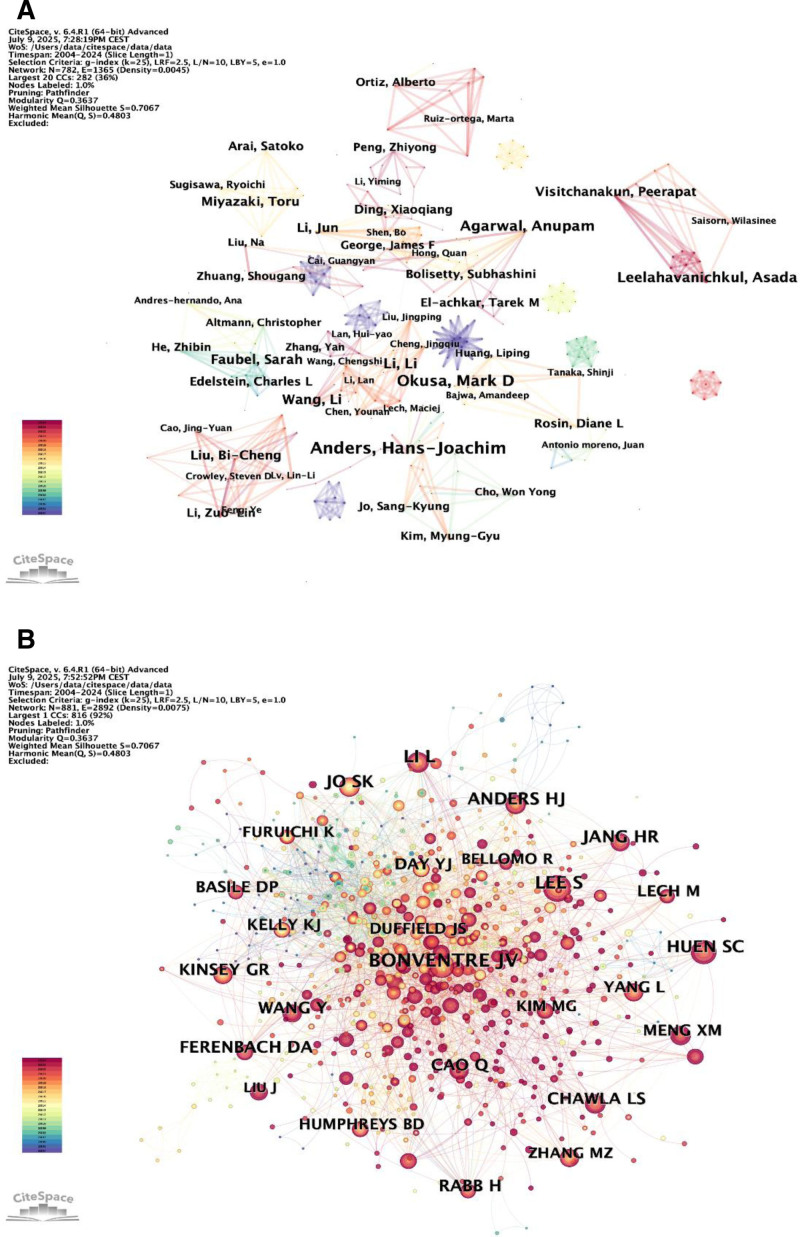
Author co-authorship analytic network generated by CiteSpace. (A) Authors co-occurrence map of annual publication count. (B) Co-occurrence map of cited authors.

Upon careful examination of authorship data, it was determined that a total of 782 authors contributed to publications related to macrophages in AKI. Within this group, 8 authors had a publication record of at least 9 papers. Using the CiteSpace program, we created visual maps to illustrate the co-authorship relationships. A minimum threshold of 1 document per author was established for inclusion in the analysis. The visual representation employs circles whose sizes correspond to the number of papers published by each participant. Additionally, various colors are used to indicate different time periods and to identify distinct clusters. The thickness of the lines connecting the circles represents the degree of collaboration. Among them, Okusa, Mark D, and Charles L. Edelstein demonstrated the most closely interconnected collaboration partnerships (Fig. 11A). We found that research in this field was predominantly led by scholars from Europe and America. It is worth noting that the collaboration network remained fragmented, with only a few teams establishing stable partnerships (such as the Okusa–Edelstein group and the Ortiz–Ruiz–Ortega group), while the majority of researchers worked in isolation. This highlights the need to foster cross-team and international collaboration to better understand the complex mechanisms of macrophages in AKI. Furthermore, Hans-Joachim Anders and Mark D. Okusa occupied the top 2 positions, while Agarwal, Anupam, and David J. Nikolic-Paterson were tied for 3rd place, underscoring their significant contributions to the field of macrophages in AKI. Table 5 lists the top 5 most productive authors in this domain and summarizes their current research areas.

**Table 5:** Annual publication counts for the top 5 authors for the period 2004 to 2024.

Rank	Author name	Count	Centrality	Year	Research areas
1	Anders, Hans-Joachim	17	0	2009	Clinical Immunology, Internal Medicine (General Medicine), Nephrology
2	Okusa, Mark D	14	0	2008	Inflammation, fibrosis, progression of kidney disease
3	Agarwal, Anupam	12	0	2015	Acute kidney injury, critical care nephrology, progression of kidney disease
4	Nikolic-Paterson, David J	12	0	2011	Macrophage, Renal Pathology, Kidney disease
5	Li, Li	11	0	2008	–

Table 6 delineates the top 10 researchers based on the frequency of citations. Each of these top 10 authors was cited over 100 times. The most frequently cited author was Bonventre JV (260 citations), followed by Lee S (200), Li L (185), Jo SK (149), and Huen SC (148). Additionally, a density map was created using the 881 authors with at least 1 co-citation, effectively illustrating high-frequency cited authors based on circle size. Over time, the color scheme transitions from green to red. As shown in Figure 11B, Bonventre JV’s region is the darkest red, indicating that this author was the most cited in the early stages of this field. Over time, this prominence is progressively overtaken by Huen SC, Cao Q, and Ferenbach DA.

**Table 6:** Top 10 most cited authors for the period 2004 to 2024.

Rank	Author	Citations	Year
1	Bonventre JV	260	2005
2	Lee S	200	2009
3	Li L	185	2008
4	Jo SK	149	2006
5	Huen SC	148	2015
6	Jang HR	145	2010
7	Anders HJ	136	2004
8	Rabb H	127	2004
9	Chawla LS	125	2013
10	Cao Q	122	2012

## 4. Discussion

AKI is characterized by a rapid decline in renal function and is one of the most common complications in critically ill patients. Moreover, AKI is a significant factor contributing to the increasing incidence of CKD and end-stage renal disease worldwide.^[28]^ The pathogenesis of AKI is multifaceted, involving various modes of cell death, including pyroptosis and ferroptosis.^[29,30]^ Inflammatory and immune responses, mitochondrial dysfunction, metabolic reprogramming, and microcirculatory disturbances are all recognized as key mechanisms contributing to AKI.^[31,32]^ Activation of the innate immune system (such as the formation of neutrophil extracellular traps, macrophage polarization, and complement activation) plays a central role in both the initiation and progression of AKI, as well as its transition to CKD.^[33]^ Mitochondrial dysfunction further exacerbates disease progression, while metabolic reprogramming leads to increased glycolysis in renal TECs, which can promote inflammation.^[34]^ Targeted interventions aimed at these underlying mechanisms may be crucial for the prevention and effective management of AKI. As key mediators in the activation and regulation of molecular and signaling pathways, macrophages play a significant role in the inflammatory processes associated with AKI development.

Macrophages, which are innate immune cells, play a crucial role in host defense and tissue homeostasis.^[35,36]^ They are involved in the injury, repair, and fibrosis processes induced by various diseases leading to AKI.^[37]^ This indicates that macrophages are engaged in all stages of AKI injury and repair responses, making them a potential key therapeutic target. Bibliometric analysis is a reliable and practical tool for analyzing literature. It enables the identification of key features and trending topics within a body of literature, facilitating a rapid understanding of new fields.^[38]^ Hence, using the bibliometric analysis approach may provide a thorough comprehension of the topic, thereby greatly aiding future study and therapeutic treatment. This research presents a bibliometric analysis of macrophages in AKI to investigate the current state of growth.

We conducted a bibliometric analysis and visualization of 1483 papers on macrophages in AKI from the WoS database as part of our research. Over the last 2 decades, there has been a general increase in the amount of macrophages mentioned in research papers related to AKI. However, over the past 3 years, this number 1st declined and then experienced a rapid increase. It demonstrates that ferroptosis, Evs and macrophage have attracted more and more attention in this field.

The US Department of Veterans Affairs has published the largest number of publications among organizations. Concerning countries, China and the United States lead the way in the number of publications, closely followed by Japan and Germany. AKI occurs in about 13.3 million people per year, 85% of whom live in the developing world, is thought to contribute to about 1.7 million deaths every year.^[39]^ Yang Li et al discovered that AKI has become a major medical burden in China, with a high proportion of patients going undiagnosed and untreated.^[40]^ As a consequence, China’s high incidence of AKI and its emphasis on medical research may contribute to its dominant position. Nevertheless, it also highlighted the requirement for significant improvements in the research quality in China. Out of the 10 journals that were mentioned, Kidney International was quoted the most frequently. Furthermore, in 2025, the journal’s latest impact factor reached 12.6, indicating the superiority in quantity and quality. Moreover, it is worth noting that 8 out of the top 10 journals that have published the most articles on macrophages in AKI are American journals. This observation highlights the significant interest and dominant role of the United States in this particular research area.

The cluster analysis of keyword co-appearance and reference co-citation resulted in the identification of the top 9 clusters, labeled as clusters 0 to 9. These clusters are shown in Figure 5B and Figure 8A and B, respectively. These clusters correspond to the keywords or concepts that have the highest frequency of occurrence in the literature, thereby indicating the areas of greatest interest at a certain time. Cluster 0 “chronic kidney diseases,” Cluster 1 “septic shock,” and Cluster 2 “acute kidney injury” are the most prevalent directions in the current research. This shows the great concern about CKD, the involvement of septic shock in AKI, and the pathological process involved. Cluster 3 “renal injury,” cluster 4 “macrophage activation syndrome,” such as a new research topic represents research into the macrophage activation and renal injury, as well as other areas of the new extension. Furthermore, during the initial period (2004–2010), the study mostly concentrated on investigating fundamental processes, such as ischemia/reperfusion damage, oxidative stress, apoptosis, and so on. During the middle stage (2011–2017), research hotspots have expanded considerably, with particular emphasis on the infiltration of macrophages and their alternative activation, specifically toward the M2 phenotype, which has emerged as a key research theme. Additionally, immune regulatory mechanisms are gaining increasing attention, including the roles of T cells, lipopolysaccharide-induced inflammatory responses, nitric oxide signaling, as well as associated metabolic and signaling pathways. Notably, diagnostic approaches are beginning to attract scholarly interest, potentially reflecting a growing demand for clinical translation. In recent years (2018–2024), cutting-edge research on macrophages in AKI has primarily focused on their critical roles in renal repair and regeneration, particularly through the pro-repair macrophage phenotype, as well as their involvement in the progression from AKI to CKD, including conditions such as diabetic nephropathy. Therefore, identifying key regulatory targets that influence macrophage polarization and function has become central to the development of novel therapeutic strategies.

Cluster 0 “chronic kidney diseases,” and cluster 1 “septic shock” have drawn much attention, particularly cluster 0 “chronic kidney diseases.” Currently, the process by which AKI develops into CKD is intricate and lacks complete comprehension. The possible ways in which AKI might lead to CKD are continuous stimulation of fibroblasts, stoppage of the G2/M cell cycle, senescence of tissue cells, damage to mitochondria, chronic inflammation, and alterations in epigenetic patterns.^[41–43]^ Among them, macrophages play an important role in AKI to CKD through interacting with multiple factors. Based on their activation state, function, and secreted components, researchers have categorized macrophage phenotypes into 2 main types: M1 and M2 macrophages. Multiple studies have shown that M1-type macrophages are inflammatory cells that play a role in the early stages of AKI. On the other hand, M2-type macrophages, depending on their specific subtype, have the ability to reduce inflammation and facilitate the healing or scarring of renal tissue after AKI.^[35,44,45]^ Macrophages serve as the primary effector cells in the inflammatory response associated with AKI. Various stimulatory molecules generated in different forms of AKI can activate macrophages, leading to their differentiation into distinct functional subsets that perform specific physiological roles. For instance, inflammatory chemokines can stimulate macrophage activation, and a deficiency in these chemokines may attenuate the progression of AKI by inhibiting macrophage activation.^[46]^ Moreover, histiocytes and macrophages interact through the release of EVs, whereby stressed histiocytes can influence neighboring macrophages, promoting their activation and contributing to tissue damage.^[47]^ Additionally, extracellular matrix proteins have been shown to induce macrophage recruitment and enhance inflammatory responses.^[48]^ During the progression of AKI, activated macrophages migrate to the site of injury and exert their effects by releasing various bioactive molecules, which either exacerbate renal damage or facilitate tissue repair. For example, pro-inflammatory cytokines such as IL-6, TNF-α, and IL-12 are predominantly secreted by M1-type macrophages and play crucial roles in amplifying inflammatory responses, particularly in both infectious and noninfectious inflammatory conditions. These cytokines can intensify inflammation and tissue injury, while inhibitors of pro-inflammatory cytokines have demonstrated protective effects by suppressing inflammation and oxidative stress. Furthermore, several molecules, including human lipocalin-2, macrophage migration inhibitory factor, and CD163, have been identified as key regulators in the pathogenesis of AKI.^[49,50]^ Macrophages also contribute to disease progression through the activation of signaling pathways such as nuclear factor-κB and retinoic acid signaling pathways.^[16,51]^

Macrophage-mediated repair following injury disrupts key mechanisms that contribute to the progression of AKI to CKD. Hui Yao Lan et al proposed that in cases when renal damage persists and worsens, macrophages have the ability to transform into profibrotic and myofibroblast phenotypes via a mechanism known as MMT. MMT cells have a phenotype that promotes inflammation and fibrosis, perhaps contributing to the progression of AKI-to-CKD. This phenotype is closely controlled by the TGF-β/Smad3 signaling pathway.^[37]^ In addition, Yao et al utilized single-cell RNA sequencing technology to construct a comprehensive profile of macrophage dynamics throughout the progression of AKI-CKD. They identified that CD38 high-expression (CD38hi) macrophage subsets were closely associated with renal fibrosis. The study further investigated the origin and functional mechanisms of CD38hi macrophages in the context of AKI-CKD progression, and validated the therapeutic potential of targeting CD38 as a strategy to mitigate renal fibrosis.^[52]^ Macrophages play dual roles as both “good guys” and “bad guys” in AKI. They can regulate macrophage activation through signaling pathways such as nuclear factor-κB, MAPK, and JAK/STAT, thereby promoting the polarization of M1 macrophages and triggering inflammatory responses. At the same time, macrophages may exacerbate kidney damage or contribute to tissue repair after AKI by releasing pro-inflammatory cytokines, macrophage extracellular traps, and other mediators. Although these signaling pathways and molecules represent potential therapeutic targets for modulating macrophage function in the prevention and treatment of AKI, the potential interactions and crosstalk among them remain to be further elucidated.

Cluster 2 “septic shock” is another hotspot of macrophages in AKI. Septic shock is a severe clinical syndrome caused by microorganisms and their toxins or other harmful products, accompanied by systemic signs of shock.^[53]^ Sepsis can lead to AKI, referred to as sepsis-associated acute kidney injury (S-AKI), which is a serious complication significantly contributing to morbidity and mortality in septic patients. In S-AKI, the underlying pathophysiology involves dysfunction and damage across multiple cell types, including macrophages, vascular endothelial cells, and renal TECs, as well as complex interactions among them.^[54]^ Among these, macrophages have garnered significant attention. Previous studies have demonstrated that macrophages exert protective immunomodulatory effects during septic AKI, thereby ameliorating tissue injury. The research team led by Steven D. Crowley investigated the functional role of the F4/80^hi^ macrophage subset in septic AKI. Specifically, following sepsis, F4/80^hi^ macrophages polarize toward an anti-inflammatory phenotype and exhibit elevated expression of anti-inflammatory genes such as Socs3, Il1r2, and Il1rn. Notably, Il1rn encodes IL-1 receptor antagonist (IL-1ra), which inhibits the activation of the IL-1 signaling pathway. This inhibition subsequently suppresses IL-6 production by endothelial cells, thereby limiting the propagation of inflammation and ultimately exerting a protective effect in septic AKI.^[55]^ Huang et al demonstrated that macrophages alleviate renal inflammation via MARCHF1- and MARCHF8-mediated autophagic degradation of TARM1, thereby ameliorating AKI.^[56]^ Furthermore, Liu et al reported that macrophages engineered through direct incubation with TPA2PyPh effectively eradicated bacteria and reduced bacterial load in a murine sepsis model.^[57]^ Collectively, these findings highlight the therapeutic potential of macrophages in the treatment of S-AKI. By using cluster analysis and the timeline view of keywords, we can determine that septic shock is not only the most prominent cluster but also a rapidly developing research frontier in recent years. To sum up, an in-depth understanding of the mechanism of septic shock may become a hot topic in the future prevention and treatment of AKI.

In our analysis of the most frequently cited literature and authors, we identified Tang PMK’s article, titled “Macrophages: versatile players in renal inflammation and fibrosis” as having the highest number of citations. This study primarily investigated the central and multifaceted roles of distinct macrophage phenotypes in driving the initiation, persistence, and subsequent fibrotic progression of renal inflammation. Following this, Huen SC’s 2017 article, “Macrophages in Renal Injury and Repair,” published in the *Annual Review of Physiology*, similarly highlights the critical importance of macrophages in kidney injury and repair. These articles primarily investigate the influence of macrophages on renal inflammation, fibrosis, and injury repair. Notably, they all emphasize the distinctive roles of various macrophage phenotypes in the inflammatory and reparative processes associated with kidney diseases. Huen SC has also contributed numerous highly cited articles, particularly in 2015 and 2017. The works of Lee S and Bonventre JV are similarly influential. Research by Han HI, Lever JM, Ronco C, and colleagues has further enriched the understanding of the role of macrophages in AKI, with studies becoming increasingly detailed and diverse.

In our analysis of the most influential authors, we identified the top 5 most prolific researchers in the field of macrophages in AKI and their respective research areas. Monitoring these authors and journals will help 1 stay abreast of the latest developments in this domain. A comparative analysis of the top 10 most cited articles reveals a shift in research focus and hotspots regarding macrophages and AKI in recent years. For instance, early studies extensively explored the complex roles of macrophages in the onset and progression of AKI, particularly emphasizing the critical roles of different macrophage phenotypes and activation states in kidney inflammation, repair, and fibrosis. In contrast, more recent research has concentrated on a deeper understanding of various aspects of AKI, highlighting the significant contribution of inflammatory responses to kidney damage in AKI and the involvement of the innate and adaptive immune systems. Furthermore, critical care nephrology has gradually attracted increasing attention. The importance of modulating immune cells to enhance the understanding of AKI has been underscored, providing a foundation for developing new diagnostic and therapeutic targets.

A robust inflammatory response is a hallmark of AKI and a critical factor in exacerbating kidney damage.^[58–60]^ The immune system is inherently involved in any inflammatory response. During the processes of kidney injury and repair, immune cells, cytokines, and the complement system directly participate in and regulate the inflammatory response.^[59]^ Among immune cells, macrophages are particularly well-known. Our research further confirms the increasingly prominent role of immunoinflammatory studies focusing on macrophages in the context of AKI. Research has demonstrated that a defect in the E-prostanoid 3 receptor, particularly in myeloid cells, hinders inflammation and disrupts the cycle of necrotic inflammation, leading to an improvement in ischemic AKI.^[61]^ The study has discovered that phosphoglycerate mutase 5 is upregulated in TECs. By mediating the dephosphorylation of the pro-apoptotic protein Bax at serine 184, phosphoglycerate mutase 5 activates the cGAS-STING signaling pathway, thereby promoting the inflammatory response in AKI.^[62]^ In addition, Li Yang et al employed single-cell sequencing to identify disease-associated macrophage subpopulations, specifically S100A8/A9^+^, during ischemia/reperfusion injury. Additionally, they confirmed the infiltration of S100A8/A9^+^ macrophages in the kidneys of human AKI patients and established a correlation between these macrophages and tissue damage.^[63]^ This suggests that future research on AKI should prioritize investigating the role of macrophages, particularly in the context of modulating immune and inflammatory responses to improve AKI outcomes. In addition, targeted therapeutic strategies focusing on specific mechanisms of AKI have garnered significant clinical interest. Numerous studies have investigated the therapeutic potential of targeted therapy agents in AKI treatment. For instance, Tocilizumab, a monoclonal antibody that targets the IL-6 receptor, Ravulizumab, and Eculizumab, along with mitochondria-targeted antioxidant peptides such as SS-31, and Ferrostatin-1, which inhibits programmed cell death, have been utilized in relevant research.^[64–67]^ Furthermore, stem cell-based therapies and regenerative medicine have demonstrated considerable promise in treating AKI. Additionally, advancements in artificial intelligence and machine learning models have enhanced risk stratification, precise prevention, remote monitoring, and early warning systems for AKI.^[68,69]^ Collectively, these developments indicate that AKI research is progressing toward earlier intervention, greater precision, and a mechanistic understanding. Improved insights into the pathogenesis of inflammation, mitochondrial dysfunction, macrophage regulation, and various modes of cell death have led to the emergence of numerous promising targeted therapeutic approaches.

This paper analyzes the research trends and hotspots of macrophages in AKI, providing valuable insights for researchers. By understanding current trends, researchers can avoid outdated topics, minimize redundant efforts, and reduce the waste of project funds. Additionally, identifying research hotspots will enable researchers to optimize and innovate their study designs, thereby enhancing the feasibility and impact of macrophage research in AKI. At the same time, this study systematically presents the temporal evolution of research on macrophages in AKI. By reviewing the historical, current, and future trends in macrophage research within the context of AKI, this study lays the foundation for precise prevention and treatment of AKI, and provides significant reference value for researchers in this field. Ultimately, this will benefit a greater number of patients, thereby reducing the global medical burden and associated economic pressures of preventing and treating AKI.

## 5. Conclusion

Based on bibliometric analysis, we have observed a significant increase in the number of publications over the past year, suggesting a growing interest in this research area. However, further observation and validation are required to confirm this trend. China and the United States are the primary contributors to research in this field. Meanwhile, Japanese and German scholars have also played a significant role in advancing the study of macrophages in AKI. Since 2018, research on ferroptosis, EVs and repair and regeneration has been particularly active. Additionally, over the past 3 years, there has been increasing attention on studies related to systemic inflammation in AKI. Furthermore, several neglected potential research topics warrant further attention and in-depth study. Our findings summarize the current state of macrophages in AKI research and have important implications for future research direction.

## 6. Limitation

Initially, it is important to note that not all papers from the databases were considered for inclusion in the analysis. Specifically, the data used in this study were only obtained from the WoS database. Furthermore, we exclusively selected studies and review papers that were authored in the English language. Articles published in languages other than English or those that were not research or review articles were deliberately excluded from our study, resulting in some exclusions. Furthermore, the data was acquired using bibliometric software that relies on machine learning algorithms. This methodology has the potential to introduce bias into bibliometric research and subsequent discussions.

## Author contributions

**Conceptualization:** Hanlu Wang.

**Data curation:** Chengmin Ma.

**Formal analysis:** Hanlu Wang

**Funding acquisition:** Xiangyu Zhang, Lingling Bao, Xueyan Bian.

**Investigation:** Xiangyu Zhang, Qiao Zhou, Sizeng Bao, Chengmin Ma, Lingling Bao, Jianwei Ma, Yanping Wang.

**Project administration:** Xiangyu Zhang, Qiao Zhou, Sizeng Bao, Chengmin Ma, Lingling Bao, Jianwei Ma, Jiancheng Huang.

**Resources:** Hanlu Wang

**Software:** Sizeng Bao, Yanping Wang.

**Supervision:** Yanping Wang, Xueyan Bian

**Validation:** Jiancheng Huang, Xueyan Bian

**Visualization:** Jiancheng Huang

**Writing – original draft:** Xiangyu Zhang.

**Writing – review & editing:** Xueyan Bian
